# Depression and its relationship to physical activity, sedentary behaviour, and body mass index (BMI) in Malaysian adolescents: a cross-sectional study

**DOI:** 10.1186/s13690-025-01729-7

**Published:** 2025-10-01

**Authors:** Norhafizah Sahril, Norliza Shamsuddin, Wan Sarifah Ainin Wan Jusoh, Norlaila Hamid, Ahmad Ali Zainuddin, Noor Ani Ahmad

**Affiliations:** Institute for Public Health, National Institutes of Health, Ministry of Health Malaysia, Block B5, No 1, Jalan Setia Murni U13/52, Seksyen U13, Setia Alam, Shah Alam, 40170 Selangor Malaysia

**Keywords:** Depression, Physical activity, Sedentary behaviors, Obesity, Adolescents, NHMS

## Abstract

**Introduction:**

Adolescence signifies a crucial developmental period characterized by heightened susceptibility to prevalent health issues, including obesity and mental health disorders. This research aims to examine the association between Body Mass Index (BMI), physical activity, sedentary behaviour, and depression among adolescents.

**Methods:**

Data from the 2022 National Health and Morbidity Survey (NHMS): Adolescent Health Survey was analysed using a nationwide cross-sectional approach with a two-stage stratified random sampling method. The survey included a representative sample of secondary school students. Depression levels were evaluated using the Patient Health Questionnaire (PHQ-9), with a score of 10 or above indicating depression. Body Mass Index (BMI) was assessed using anthropometric measurement (weight and height) and interpreted using WHO 2007 Growth Reference Data for 5–19 years. A total of 33,523 school-attending adolescents participated in the survey, yielding a response rate of 89.4%. For the purpose of this study, however, analysis was limited to data from 33,407 respondents who fully completed all PHQ-9 questions. Descriptive and complex sample logistic regression analyses were performed using SPSS version 26.0.

**Results:**

The prevalence of depression among Malaysian adolescents was 26.9%. Multiple logistic regression analysis indicated that female adolescents (AOR: 2.66, 95% CI: 2.46, 2.87) and those in higher age exhibited a higher likelihood of experiencing depression. Conversely, Chinese (AOR: 0.58, 95% CI: 0.48, 0.69) and Indian adolescents (AOR: 0.67, 95% CI: 0.54, 0.82) were less likely to experience depression. Depression was associated with increased sedentary behaviour (AOR: 2.01, 95% CI: 1.85, 2.18) and obesity (AOR: 1.12, 95% CI: 1.01, 1.23).

**Conclusion:**

The findings indicate that one in four Malaysian adolescent experiences depression, with significant risk factors including gender, age, high levels of sedentary behavior, and obesity. These findings emphasize the importance of public health strategies that address these critical factors and depression by encouraging active lifestyles, reducing obesity, and incorporating mental health interventions.

**Table:** 

**Text box 1. Contributions to the literature**
• Provides nationally representative evidence on the prevalence and correlates of depression among Malaysian adolescents post-COVID-19.
• Highlights the significant roles of sedentary behaviour and obesity, rather than physical inactivity alone in influencing adolescent mental health.
• Offers insight into ethnic and gender disparities in depression, contributing to a culturally nuanced understanding of mental health in multi-ethnic settings.
• Emphasizes the importance of integrating physical and mental health strategies in adolescent public health programs.

## Background

Depression is a widespread mental health disorder among adolescents globally, representing a major public health issue [1, 2]. Adolescence, defined as the period between ages 10 and 19, is a critical developmental stage marked by significant changes across physical, cognitive, emotional, and social domains. These transitions can greatly influence young people’s health and overall well-being [3]. Current estimates indicate that around 21.3% of children and adolescents experience depression or depressive symptoms, a figure that has steadily increased over time [4]. This trend is especially pronounced in high-income countries, where the Global Burden of Disease study has highlighted concerning rates of depression among individuals aged 10 to 24, with females being disproportionately affected [5, 6].

Rising rates of adolescent depression have been attributed to a variety of risk factors and are associated with adverse outcomes, including impaired social functioning and poor academic performance [2, 7]. Research shows that children and adolescents with depression often face challenges in school, such as difficulties with concentration, motivation, and achievement [8]. Furthermore, depression is associated with reduced school engagement, which can negatively affect educational outcomes and limit future opportunities [7, 8]. The consequences of adolescent depression are substantial. Many affected individuals experience social withdrawal and isolation, which can strain relationships and weaken their support systems [7]. Depression can also hinder a young person’s ability to engage meaningfully with peers and family, resulting in further social disconnection [9]. This tendency toward withdrawal and difficulty expressing emotions often contributes to relationship breakdowns and a deepened sense of loneliness. Beyond social effects, depression significantly undermines overall well-being in children and adolescents [10]. It can lead to a loss of interest in previously enjoyable activities, feelings of hopelessness and worthlessness, and a diminished sense of purpose and meaning in life.

Research indicates that adolescents with obesity are more likely to experience depression, exhibiting significantly greater psychological distress than those of normal weight [11]. Depression has been associated with higher BMI, with one study attributing 7.7% of BMI variation to depression scores [12]. Moreover, the prevalence of obesity is associated with an increased risk of depression, as highlighted by a meta-analysis reporting an odds ratio of 1.33 for depression among individuals with obesity [13]. Another study in adolescents found that obesity is similarly associated with increased depressive symptoms and a higher risk of clinical depression compared to their normal-weight peers [14, 15]. Longitudinal studies show that obesity in adolescence can predict later depressive disorders [16], with one large study finding that adolescents with severe obesity had more than twice the odds of reporting depressive symptoms [17]. Meta-analyses reinforce these findings, with one reporting a 43% higher likelihood of depression in adolescents with obesity [18]. Although primarily studied in adults, metabolically unhealthy obesity also appears relevant in youth. Notably, individuals with metabolically unhealthy obesity are identified as being at the greatest risk of developing depression [19].

Sedentary behaviour, particularly in adolescents, has become increasingly associated with adverse mental health outcomes, including depression. Recent meta-analyses and systematic reviews consistently highlight a positive association between increased sedentary time, especially screen-based activities, and a higher risk of depressive symptoms in this age group [20, 21]. Extended screen time, often involving social media, gaming, or television, has been associated with decreased mental well-being, increased frustration, and impaired focus [22, 23]. Excessive screen use, beyond its impact on mood, has also been associated with a cluster of health concerns including obesity, potential cognitive impairments, and various psychological challenges in adolescents [24, 25]. The dose-response relationship is a critical aspect, with research indicating that exceeding certain thresholds of daily screen time (e.g., more than 2–3 h of recreational screen use) is more strongly associated with clinically relevant depressive symptoms and lower psychological well-being [22, 26]. High levels of overall sedentary behavior, not limited to screens, are frequently reported to correspond with lower mental well-being and life satisfaction [22, 27]. In addition, physical inactivity is a well-established risk factor for depression [13, 28]. In contrast, regular physical activity is recognized for its protective effects against both obesity and depression, enhancing mood and reducing stress [24]. Engaging in moderate to vigorous physical activity can help mitigate the harmful effects of a sedentary lifestyle, promoting overall physical and mental health [24]. Physical exercise also contributes to improved cognitive function and emotional stability, providing a buffer against depression [22, 29]. Despite these benefits, many adolescents do not meet the recommended levels of physical activity, highlighting a critical public health concern. Addressing this gap is essential for improving adolescent health in the digital age [30, 31].

One notable study conducted among Malaysian children aged 8 to 11 years, drawn from three primary schools in the east coast region of the country, identified a significant association between body mass index (BMI) and the presence of depressive symptoms. The findings revealed that the highest prevalence of obesity measured at 42.1% was observed among children who exhibited signs of depression, highlighting the possible psychological burden associated with excess weight even in younger populations [32]. These results underscore the importance of addressing both the physical and emotional dimensions of health in school-aged children. This concern is further contextualized by data from the National Health and Morbidity Survey: Adolescent Health Survey 2017, which found that only 19.8% of Malaysian adolescents met the recommended levels of physical activity. The overwhelming majority were insufficiently active, pointing to a sedentary lifestyle trend that may be contributing not only to rising obesity rates but also to worsening mental health outcomes among youths [33]. Supporting this connection, another Malaysian study involving secondary school students from five randomly selected schools in Kuala Lumpur reported a significant association between low physical activity and elevated depressive symptoms. The researchers emphasized that students with limited engagement in physical activities were more likely to report experiencing symptoms of depression, suggesting a bidirectional relationship between physical inactivity and poor mental health during adolescence [34]. Results from the same study also found significant correlations between problematic internet usage, an element of sedentary behavior and higher levels of depressive symptoms [34]. However, it is important to note that not all local studies have arrived at consistent conclusions. For example, a separate investigation involving a large and representative sample of 1,747 adolescents aged 13 to 14 years from six schools located in the southern region of Malaysia yielded more nuanced results. While this study found that over half of the adolescents reported low levels of physical activity and that approximately 40% exhibited symptoms of depression, it did not identify a statistically significant association between these two variables [35]. This divergence in findings suggests that the relationship between physical activity, obesity, and mental health may be influenced by a variety of contextual and individual factors, including socio-demographic background, school environment, and family dynamics.

Despite some evidence suggesting associations between obesity, physical activity, and depression, there is a lack of research examining how specific factors, such as BMI, sedentary behaviour, and physical activity, interact to influence depression among Malaysian adolescents. Therefore, this study aims to explore these associations, offering insights that may guide targeted interventions to address depression and improve health outcomes in this population.

## Methods

### Study design, sample size and sampling design

The National Health and Morbidity Survey (NHMS) has been an important platform for monitoring the health of the population in Malaysia. The objectives of the NHMS are to provide the community-based data on the pattern of common health problems, health service utilisation and health expenditure in the community. Findings of the NHMS support the Ministry of Health to review the priorities and activities of the health programme, to plan for future allocation of resources and to evaluate the impact of strategies. NHMS has been conducted every year since 2011. The focus area for each year is decided by the Ministry of Health Malaysia.

This study utilized data from the 2022 National Health and Morbidity Survey (NHMS): Adolescent Health Survey, which employed a cross-sectional design to assess Malaysian secondary school students aged 13 to 17 years. The survey was conducted between June and July 2022. The sample size was calculated to ensure both statistical reliability and representativeness of the findings, while also considering budgetary constraints. It was determined based on the objectives of each survey module, using the standard formula for estimating a single proportion. Several factors were considered in the calculation, including prevalence rates from the previous Adolescent Health Survey (AHS) 2017, a margin of error ranging from 0.01 to 0.05, and a 95% confidence interval. Adjustments were made based on the total target population, using 2021 enrolment data of school-going adolescents, to ensure sufficient precision in estimating the prevalence of the specified health conditions. These adjustments included applying a design effect of 2 and accounting for the complex sampling design, including a 20% non-response rate. As a result, the final required sample size for adolescents at national and state level were 36,000 and 2,250 respectively.

A multistage stratified cluster sampling method was used, and it involved two stages. In the first stage, secondary schools were randomly selected from a comprehensive list of eligible schools across Malaysia, with a total of 240 schools chosen. The selection process used probability proportional to enrolment (PPS) across Forms 1 to 5. In Malaysia, school grade levels are referred to as Forms. Specifically, Form 1 corresponds to age 13, Form 2 to age 14, Form 3 to age 15, Form 4 to age 16, and Form 5 to age 17. In each state, 16 schools were selected, while eight schools were selected from the smaller federal territories of Labuan and Putrajaya. In the second stage, classes were selected using systematic probability sampling with a random starting point. All classes from Forms 1 to 5 within the selected schools were included in the sampling frame, and students from the chosen classes were invited to participate, contingent upon obtaining written parental consent. The inclusion criteria for the study were students aged 13 to 17 years, while exclusion criteria included students who were absent on survey days or lacked parental consent. Trained researchers provided instructions and explanations for completing the self-administered questionnaires. A total of 33,523 school-attending adolescents participated in this survey with a response rate of 89.4%.

Ethical approvals were obtained from the Medical Research and Ethics Committee (MREC) of the Ministry of Health Malaysia (NMRR-21-157-58261), and permission to conduct the study was secured from the Ministry of Education at the national, state, and school levels. Detailed methodologies for the National School-Based Health Survey 2022 have been published elsewhere [36].

### Measures

#### Depression

The variable examined in this study, depression, was assessed using the Patient Health Questionnaire-9 (PHQ-9), a self-report instrument designed to measure the presence of depressive symptoms based on the nine diagnostic criteria from the Diagnostic and Statistical Manual-IV (DSM-IV). The validated Malay version of the PHQ-9 was employed in this study to identify depression among participants [37]. The Malay version of the PHQ-9 was found to have good internal reliability (Cronbach’s alpha = 0.70) [37]. This instrument is derived from the original English version developed by Kroenke, Spitzer, and Williams in 2001 [38].

The questionnaire assesses symptoms experienced in the two weeks preceding its completion. Each of the nine items provides four response options: “not at all,” “several days,” “more than half the days,” and “nearly every day,” with corresponding scores ranging from 0 (no occurrence) to 3 (almost daily occurrence). The total score ranges from 0 to 27. In this study, participants with a total score of 10 or higher were classified as experiencing depression [37].

#### Independent variables

The National School-Based Health Survey 2022 employed a self-administered questionnaire adapted from the World Health Organization’s Global School-based Student Health Survey, available in two languages Malay and English. In this study, the independent variables comprised socio-demographic factors, including sex, age, and ethnicity. Additional risk factors examined were physical activity levels, sedentary behavior, and BMI. Physical activity was defined as engaging in at least 60 min of physical activity per day, for at least of five days per week, summing up all the time spent in any kind of physical activity each day [36]. Sedentary behaviour referred to spending three hours or more per day sitting for leisure activities, such as watching television, playing computer games, talking to friends, or surfing the internet [36]. BMI was calculated using anthropometric measurements of weight and height, then categorised based on the World Health Organization (WHO) 2007 Growth Reference for individuals aged 5 to 19 years [39]. This classification uses BMI-for-age z-scores that are specific to both age and sex to categorize individuals into four groups: underweight, defined as a BMI-for-age z-score below minus two standard deviations; normal weight, defined as a BMI-for-age z-score between minus two and plus one standard deviations; overweight, defined as a BMI-for-age z-score above plus one standard deviation; and obesity, defined as a BMI-for-age z-score above plus two standard deviations [39].

### Data analysis

The data analysis included only the data from 33,407 respondents who fully completed all PHQ-9 questions. Descriptive statistics, involving the calculation of frequencies, were used to summarize the characteristics of the study population. The Chi-square test was used to determine the bivariate relationship between the studied variables and depression. To investigate the associations between depression and various independent variables, both univariable and multivariable logistic regression analyses were conducted. Results are reported as crude and adjusted odds ratios (OR) with 95% confidence intervals (CI). Variables with a *p*-value less than 0.25 in the univariable analysis were included in the multivariable model, and statistical significance was determined at a *p*-value below 0.05. To ensure the appropriateness of the logistic regression model, diagnostic testing using a classification table was conducted to assess its goodness of fit. In this study, the classification table indicated an accuracy of 80.5%, which was used as part of the evaluation process. Additionally, a two-way interaction analysis was performed among all independent variables, revealing no significant interactions (*P* > 0.05). The application of weighting was intended to correct for unequal selection probabilities and non-response bias, ensuring that the sample accurately represents the target population. This approach enhances the validity and generalizability of the findings at both national and state levels.

## Results

This study received responses from 33,407 individuals, with the majority being female (53.8%) and of Malay ethnicity (69.0%). Participation was fairly evenly distributed across age. Most respondents were not physically active (78.6%) and exhibited sedentary behaviour (66.8%). Regarding BMI status, the majority had a normal body weight (61.4%), followed by overweight (16.1%), obese (14.5%), and underweight (8.0%) (Table 1).

**Table 1:** Socio-demographic characteristics (*N* = 33,407)

Socio-demographic characteristic	*n*	Percentage (%)
**Sex**		
Male	15,443	46.2
Female	17,964	53.8
**Age**		
13	7177	21.4
14	6876	20.6
15	6441	19.3
16	6735	20.2
17	6178	18.5
**Ethnicity**		
Malay	23,048	69.0
Chinese	5064	15.1
Indian	1548	4.6
Other Bumiputeras	2958	8.9
Others	789	2.4
**Physically active**		
Yes	26,224	78.6
No	7147	21.4
**Sedentary Behaviour**		
No	11,065	33.2
Yes	22,274	66.8
**Weight Status**		
Normal	20,443	61.4
Underweight	2659	8.0
Overweight	5377	16.1
Obese	4841	14.5

The overall prevalence of depression among the participants was 26.9%, with 9103 individuals reporting depression, while 73.1% (24304 individuals) did not. Regarding socio-demographic characteristics, there was a statistically significant difference in depression prevalence by sex (*P* < 0.001). A higher prevalence of females (36.1%) reported depression compared to males (17.7%). Age also showed a significant association with depression (*P* < 0.001). The prevalence of depression was 22.6% for 13-year-olds, 27.2% for 14-year-olds, 27.0% for 15-year-olds, 28.4% for 16-year-olds, and 30.1% for 17-year-olds. Ethnicity was another significant factor (*P* < 0.001). The prevalence of depression among Malays was 28.7%, Chinese 20.6%, Indian 20.2%, Other Bumiputeras 30.5%, and Others 28.6%. In terms of lifestyle factors, individuals who were not physically active had a higher prevalence of depression (27.8%) compared to those who were physically active (23.5%), and this difference was statistically significant (*P* < 0.001). Similarly, sedentary behavior was significantly associated with depression (*P* < 0.001), with 31.0% of those with sedentary behavior reporting depression compared to 18.7% of those without. Finally, Body Mass Index (BMI) also showed a significant relationship with depression prevalence (*P* < 0.001). The rates of depression were 22.0% for underweight individuals, 27.2% for those with a normal BMI, 28.3% for overweight individuals, and 26.9% for obese individuals (Table 2).

**Table 2:** Prevalence of depression status by Socio-Demographic, physical activity, sedentary and body mass index (BMI)

Socio-demographic characteristic	Depression		*P*-value (X^2^)
	Yes (%)	No (%)	
**Overall**	9103 (26.9)	24,304 (73.1)	-
**Sex**			< 0.001
Male	2682 (17.7)	12,761 (82.3)	
Female	6421 (36.1)	11,543 (63.9)	
**Age**			< 0.001
13	1704 (22.6)	5473 (77.4)	
14	1882 (27.2)	4994 (72.8)	
15	1802 (27.0)	4639 (73.0)	
16	1882 (28.4)	4853 (71.6)	
17	1833 (30.1)	4345 (69.9)	
**Ethnicity**			< 0.001
Malay	6504 (28.7)	16,544 (71.3)	
Chinese	1065 (20.6)	3999 (79.4)	
Indian	328 (20.2)	1220 (79.8)	
Other Bumiputeras	977 (30.5)	1981 (69.5)	
Others	229 (28.6)	560 (71.4)	
**Physically active**			< 0.001
Yes	1697 (23.5)	5450 (76.5)	
No	7398 (27.8)	18,826 (72.2)	
**Sedentary Behaviour**			< 0.001
No	2099 (18.7)	8966 (81.3)	
Yes	6988 (31.0)	15,286 (69.0)	
**Body Mass Index (BMI)**			< 0.001
Normal	5591 (27.2)	14,852 (72.8)	
Underweight	581 (22.0)	2078 (78.0)	
Overweight	1539 (28.3)	3838 (71.7)	
Obese	1371 (26.9)	3470 (73.1)	

The multiple logistic regression analysis revealed several significant findings. It indicated that female adolescents (AOR: 2.66, 95% CI: 2.46, 2.87) and those in older age groups had a heightened likelihood of experiencing depression. Conversely, Chinese (AOR: 0.58, 95% CI: 0.48, 0.69) and Indian adolescents (AOR: 0.67, 95% CI: 0.54, 0.82) showed a lower likelihood of experiencing depression. Furthermore, depression was associated with increased sedentary behaviour (AOR: 2.01, 95% CI: 1.85, 2.18) and obesity (AOR: 1.12, 95% CI: 1.01, 1.23) (Table 3).

**Table 3:** Factor associated with depression among School-going adolescents in Malaysia

Socio-demographic characteristic	Crude OR			*p*-value	Adjusted OR			*p*-value
	Exp(B)	95% Confidence Interval			Exp(B)	95% Confidence Interval		
		Lower	Upper			Lower	Upper	
**Sex**								
Male	1				1			
Female	2.64	2.44	2.84	< 0.001	2.66	2.46	2.87	< 0.001
**Age**								
13	1				1			
14	1.47	1.32	1.64	< 0.001	1.19	1.08	1.32	< 0.001
15	1.28	1.16	1.42	< 0.001	1.13	1.00	1.27	< 0.001
16	1.27	1.13	1.42	< 0.001	1.19	1.06	1.33	0.042
17	1.36	1.22	1.51	< 0.001	1.29	1.16	1.44	0.003
**Ethnicity**								
Malay	1				1			
Chinese	0.64	0.55	0.76	< 0.001	0.58	0.48	0.69	< 0.001
Indian	0.63	0.52	0.75	< 0.001	0.67	0.54	0.82	< 0.001
Other Bumiputeras	1.09	0.92	1.30	0.318	1.09	0.92	1.29	0.295
Others	1.00	0.76	1.31	0.982	1.02	0.78	1.34	0.874
**Physically active**								
Yes	1				1			
No	1.26	1.17	1.35	< 0.001	1.05	0.97	1.13	0.208
**Sedentary Behaviour**								
No	1				1			
Yes	1.96	1.80	2.12	< 0.001	2.01	1.85	2.18	< 0.001
**Body Mass Index (BMI)**								
Normal	1				1			
Underweight	0.75	0.66	0.85	< 0.001	0.91	0.81	1.03	0.138
Overweight	1.06	0.96	1.16	0.269	1.10	0.99	1.22	0.064
Obese	0.98	0.89	1.08	0.741	1.12	1.01	1.23	0.030

Analysis was done using complex sample logistic regression analysis. Classification Table (80.5%) were used to check model fitness

## Discussion

This study identified a depression prevalence of 26.9% among secondary school students, which is lower than the 32.7% reported by Normala et al. in their study involving 1,800 Malaysian adolescents aged 13 to 17 years [40]. Both studies utilized the PHQ-9 diagnostic tool for assessing depression. However, it is worth noting that Normala et al.‘s research was limited to students from only 10 out of 37 randomly selected secondary schools in Hulu Langat, Selangor [40]. When compared to the National Health and Morbidity Survey (NHMS) 2019, which reported a national depression prevalence of 2.3% [41], the present study reveals a markedly higher rate, almost a tenfold increase. It is important to note that NHMS 2019 did not specifically focus on adolescents, which likely contributed to the lower reported prevalence. Several key factors help explain this substantial increase in depression prevalence. Firstly, the NHMS 2019 data represented the general population and was not designed to isolate adolescent-specific outcomes [41]. Adolescents often experience higher rates of mental health issues than adults due to the unique challenges of this developmental stage, including rapid biological, emotional, and social changes. This vulnerability is well-supported by global research, such as the systematic review by Polanczyk et al. (2015), which demonstrated that the 12-month prevalence of mental disorders in children and adolescents is significantly higher than in the adult population [42]. Secondly, the COVID-19 pandemic has had a profound impact on adolescent mental health [43]. The NHMS 2019 data were collected before the onset of the pandemic, whereas the current study reflects the post-pandemic period. The pandemic brought about widespread disruptions to daily life, including prolonged school closures, social isolation, reduced physical activity, economic instability, and increased screen time, all of which negatively influenced adolescent mental well-being. A meta-analysis by Racine et al. (2021) documented a significant rise in depression and anxiety symptoms among children and adolescents during the pandemic [43]. In Malaysia, several studies conducted during and after the pandemic have similarly reported heightened psychological distress in young people, attributing it to factors such as the abrupt shift to online learning, lack of peer interaction, and increased sedentary behavior [44–46]. Beyond these major influences, differences in study design, sample characteristics, and broader contextual factors such as cultural norms, geographic settings, socioeconomic status, and mental health policy frameworks may also account for variations in depression prevalence. Unlike the household-based NHMS survey, the current study focused exclusively on school-going adolescents, allowing for more targeted mental health assessments within a specific age group.

Furthermore, increasing mental health awareness and a gradual reduction in stigma partly due to public health campaigns and the pandemic’s emphasis on psychological resilience may have encouraged adolescents to report depressive symptoms more openly in recent studies. Additionally, the 26.9% depression prevalence among Malaysian adolescents reported in this study is notably higher than the 11.1% observed among Japanese adolescents [47], yet closely aligns with the 26.5% prevalence identified in a study of Bangladeshi adolescents [48]. Both international studies also employed the PHQ-9 screening tool. Discrepancies in prevalence rates across countries can be attributed to differences in demographic profiles, research methodologies, and socio-cultural contexts. Factors such as age distribution, gender ratios, healthcare accessibility, education systems, and national mental health strategies all contribute to shaping adolescent mental health outcomes. For instance, countries with comprehensive support systems and proactive mental health policies may experience lower rates of undiagnosed or untreated depression. These findings underscore the importance of context-specific public health approaches that are sensitive to the diverse needs and challenges faced by adolescents in different settings.

The finding that female adolescents are at a higher risk of experiencing depression aligns with a robust body of literature [49, 50], and understanding the pathways through which this association manifests is crucial. This increased prevalence isn’t simply a matter of gender but is influenced by a complex interplay of psychosocial and cultural factors. One significant pathway relates to parental support and behavior. In many cultural contexts, girls often face stricter parental behaviors and receive comparatively less perceived parental support than boys. This disparity can lead to feelings of inadequacy, resentment, or a lack of autonomy, which are known contributors to depressive symptoms. When adolescents, particularly girls, feel their actions are overly controlled or their emotional needs are not adequately met by parents, it can erode their self-esteem and coping mechanisms, increasing their vulnerability to depression. Furthermore, societal expectations play a substantial role. Female adolescents are frequently exposed to messages that subtly or overtly devalue their abilities and achievements compared to their male peers [51]. This can manifest in academic settings, career aspirations, or even personal attributes, leading to internalised feelings of inferiority. The constant pressure to conform to unrealistic beauty standards or traditional gender roles can also contribute to body image issues, anxiety, and ultimately, depression. These societal pressures can create a pervasive sense of not being “enough,” which is a powerful pathway to depressive states. Finally, gender and cultural contexts significantly influence the expression and recognition of depressive symptoms. Research indicates that male adolescents often delay expressing depressive symptoms until they become more severe, possibly due to societal pressures to be stoic or “strong” [52]. In contrast, females tend to report symptoms at earlier stages, which might contribute to their higher rates of diagnosis. This difference in symptom reporting doesn’t necessarily mean males experience less depression, but rather that their symptoms might be masked or manifest differently (e.g., through aggression or substance abuse). Culturally, there might be varying degrees of acceptance for emotional expression based on gender, further shaping how depression is experienced and communicated.

Malaysia is known for its rich ethnic diversity, with the population predominantly comprising Malays, Chinese, and Indians, each possessing distinct cultural traditions, social norms, and family dynamics. In our study, we observed that adolescents of Chinese and Indian heritage reported lower levels of depression compared to their Malay counterparts. This finding presents a notable contrast to some earlier studies, which suggested that Chinese and Indian populations might be more vulnerable to depression [49]. A potential explanation for this discrepancy may lie in the specific cultural strengths inherent within the Chinese and Indian communities in Malaysia. Both groups often place a strong emphasis on family cohesion and close-knit community networks, which can serve as vital sources of emotional support and psychological stability for adolescents. In these cultures, family support is not only valued but often deeply embedded in daily life, playing a central role in helping adolescents cope with stress, anxiety, and emotional distress. Additionally, the cultural frameworks within Chinese and Indian families tend to prioritize academic excellence and uphold collectivist values, where the well-being of the group takes precedence over individual pursuits. These values may provide adolescents with a structured environment, clear expectations, and a strong sense of identity and purpose, key protective factors against the development of depressive symptoms [53, 54]. A sense of belonging and meaningful contribution to the family or community can help young people feel supported, valued, and less isolated. Furthermore, traditional practices commonly observed in Chinese and Indian communities such as meditation, prayer, and participation in religious or cultural gatherings may function as effective coping mechanisms. These practices not only help adolescents manage daily stressors but may also promote emotional regulation and inner peace, reducing their susceptibility to depression [54]. Taken together, the interplay of strong family bonds, communal values, emphasis on academic and social responsibility, and engagement in culturally rooted practices appears to foster a form of cultural resilience. Within the Malaysian context, this resilience may help explain the comparatively lower prevalence of depression among Chinese and Indian adolescents relative to their Malay peers.

Our study found that older adolescents face a higher risk of developing depression, a finding consistent with another research [55]. The increasing likelihood of experiencing depressive symptoms as adolescents get older isn’t a coincidence; it stems from a combination of intensifying developmental, academic, social, and emotional changes. Grasping these contributing factors is essential for effectively addressing their mental health needs. A primary influence on this trend involves the cumulative effect of significant physical, emotional, and social changes characteristic of later adolescence. While puberty introduces hormonal shifts throughout adolescence, its later stages can bring about more complex physiological and neurological maturation that influences mood regulation and emotional reactivity [56]. Emotionally, older adolescents are developing a more refined sense of self and a greater capacity for abstract thought [57]. This can lead to increased self-reflection and a heightened awareness of existential concerns, personal flaws, or societal issues, potentially fostering rumination and negative self-perception [58]. Socially, the peer group’s influence grows stronger, and navigating complex social hierarchies, friendships, and romantic relationships becomes more demanding. This often includes more significant experiences of rejection, social comparison, or betrayal, which are potent stressors during this period [59]. Furthermore, increasing academic pressures are a major factor. As adolescents advance through high school and prepare for higher education or careers, the stakes rise significantly. The demands for academic performance escalate, with more challenging coursework, standardized tests, and the pressure to achieve good grades for college admissions or employment. This constant pressure can lead to considerable stress, anxiety, and feelings of inadequacy, particularly if perceived performance doesn’t meet expectations, directly contributing to depressive symptoms [60, 61]. Evolving family dynamics also play a crucial role. As adolescents mature, their drive for autonomy often leads to shifts in family relationships [57]. While this is a normal part of development, it can sometimes result in increased conflict with parents, a perceived reduction in parental support, or a feeling of being misunderstood [62]. The transition toward greater independence, coupled with the ongoing need for guidance and connection [63], can be a delicate balance, and disruptions in family harmony can significantly affect an older adolescent’s mental well-being [64]. Lastly, heightened emotional awareness and emotion regulation difficulties in older adolescents, while a sign of cognitive maturity, can paradoxically increase their vulnerability to depression [58, 65]. They become more capable of recognizing and labelling complex emotions in themselves and others. While this aids in empathy, it also means they can more acutely feel and process negative emotions such as sadness, hopelessness, or anger. This increased capacity for emotional processing, without fully developed coping mechanisms or adequate support systems, can lead to overwhelming emotional experiences that manifest as depressive symptoms [66]. They may also become more aware of broader societal problems, global issues, or personal limitations, leading to feelings of helplessness or despair.

The significant association between sedentary behaviour and depressive symptoms observed in Malaysian adolescents mirrors a concerning global trend. A systematic review has provided strong evidence of an association between leisure screen time and depressive symptoms, showing that adolescents who spend more than 2 to 3 h daily in sedentary activities are at greater risk for poorer mental health outcomes [67]. This dose-dependent relationship is further substantiated by a large cross-sectional study across low- and middle-income countries, which found that 3 or more hours of daily sedentary activity increased the odds of depressive symptoms by 20% [68]. The association between excessive sedentary behaviour, particularly screen time, and depression is likely multifaceted. One primary mechanism involves the displacement of physically and mentally beneficial activities. For instance, a study [69] demonstrated that reallocating sedentary time to even light physical activity significantly ameliorated depression scores in typically inactive adolescents. This suggests that the reduction in physical activity, and consequently its associated neurobiological benefits (e.g., endorphin release, improved stress regulation) and psychosocial advantages (e.g., social engagement, enhanced self-efficacy), is a critical mediating factor [70, 71]. Furthermore, the nature of screen-based sedentary activities themselves may contribute directly. A recent narrative review [22] identified a strong connection between excessive screen time and a spectrum of psychological disorders. This could be due to psychosocial influences such as increased opportunities for negative social comparison [72], cyberbullying [73], or a reduction in restorative face-to-face interactions [74], all of which are established risk factors for depression. Additionally, neurobiological processes may be implicated; for example, prolonged screen exposure can disrupt sleep patterns due to blue light and overstimulation, and sleep disturbance is both a core feature and a risk factor for depression [75, 76]. The observed correlation of prolonged sedentary behaviour with increased depressive symptoms in adults, potentially stemming from habits formed in adolescence [77], underscores the long-term implications. Mendelian randomization studies have supported a causal association between sedentary leisure activities, such as television watching, and an increased risk of depression [78]. This causal relationship is further supported by findings from a large Chinese cross-sectional study [79], which revealed that adolescents engaging in multiple unhealthy lifestyle habits, with excessive screen time being a prominent one, faced significantly amplified odds of developing depression.

The current study found a significant association between obesity and depression among Malaysian adolescents. This finding is consistent with previous Malaysian studies, which have similarly identified a strong correlation between higher body mass index (BMI) and increased depressive symptoms among school-aged children [32]. Given that approximately 37% of Malaysian adolescents are classified as obese, this high prevalence raises serious concerns about the broader psychological and emotional impacts of obesity within this population [32]. Adolescents living with obesity frequently encounter a host of psychosocial stressors that can contribute to the onset or exacerbation of depressive symptoms. These challenges often include dissatisfaction with body image, experiences of social exclusion, stigmatization, and diminished self-esteem, all of which are well-established risk factors for depression [80]. Beyond these psychological dimensions, elevated BMI has also been associated with increased reports of psychosomatic complaints, suggesting that the connection between obesity and mental health extends into physiological territory as well [81]. Obesity may influence emotional well-being not only through external social pressures but also through internal biological mechanisms such as chronic inflammation or hormonal imbalances, both of which can affect mood regulation. Further complicating this relationship are lifestyle factors that are particularly prevalent during adolescence. Patterns such as excessive screen time, sedentary behaviour, and unhealthy dietary habits are common among youth and are known to contribute independently to both obesity and poor mental health outcomes [22]. These behaviours may reinforce one another, creating a harmful cycle in which physical inactivity and poor nutrition lead to both weight gain and a decline in emotional well-being. In many cases, adolescents with depression may engage in emotional eating as a coping strategy, which in turn results in further weight gain. This cyclical pattern can deepen depressive symptoms and entrench the relationship between obesity and poor mental health over time [57]. In the Malaysian socio-cultural context, these challenges may be further intensified by cultural norms and expectations related to physical appearance. Societal attitudes that emphasize thinness or stigmatize larger body types can place added psychological pressure on adolescents struggling with obesity. These youths may experience heightened feelings of inadequacy or failure to conform to unrealistic body ideals, further compounding their vulnerability to depression and related mental health issues [58]. Taken together, the findings from this study and prior research suggest that the relationship between obesity and depression in Malaysian adolescents is shaped by a complex interplay of psychosocial, physiological, behavioural, and cultural factors. Addressing this issue effectively will require integrated strategies that not only promote healthy lifestyle habits but also foster supportive environments that reduce stigma and enhance emotional resilience among youth.

In this study, physical activity showed no significant association with depression, as the initial relationship observed in the univariable analysis was likely confounded by other factors. After adjusting for variables such as sex, age, ethnicity, BMI, and sedentary behaviour in the multivariable analysis, the association between physical activity and depression lost statistical significance. This suggests that the effect of physical activity on depression may be mediated or moderated by these other variables. It’s also possible that sedentary behaviour, especially prolonged screen time, has a stronger and more direct impact on adolescent mental health than physical activity alone. Additionally, limitations in how physical activity was measured such as relying on self-reported data without capturing intensity, duration, or context may have contributed to the lack of observed association. Therefore, the absence of a significant association in this study does not necessarily imply that physical activity has no effect on depression, but rather that its impact may be indirect, context-dependent, or obscured by measurement and confounding factors.

This study, like others, has both strengths and limitations. One of the key strengths of this study is its large, ethnically diverse participant pool, enhancing its ability to identify significant relationships and improve the reliability of the findings. Furthermore, by focusing on a population-based sample rather than a clinical one, the results are more applicable to the general adolescent population. However, a notable limitation is the cross-sectional design, which captures data at a single point in time and therefore does not allow for the establishment of causal relationships. Additionally, the reliance on self-reported data introduces the risk of information and recall bias, which may impact the accuracy of the conclusions drawn from the study.

## Conclusion

The findings reveal that approximately one in four Malaysian adolescents experiences depression, with significant risk factors including gender, age, sedentary behaviour, and obesity. These results highlight the critical need for public health strategies that address these factors, with a particular focus on integrating clinical practice guidelines for adolescent health. Further research is warranted to explore the underlying mechanisms associated with these factors and to develop targeted interventions to improve mental health outcomes among adolescents.

## Data Availability

The data that support the findings of this study are available from Ministry of Health Malaysia but restrictions apply to the availability of these data, which were used under license for the current study, and so are not publicly available. Data are however available from the authors upon reasonable request and with permission of Ministry of Health Malaysia.

## Abbreviations

BMI: Body Mass Index
NHMS: National Health and Morbidity Survey
PHQ-9: Patient Health Questionnaire 9
DSM-IV: Diagnostic and Statistical Manual-IV
